# An Automated Sitting Posture Recognition System Utilizing Pressure Sensors

**DOI:** 10.3390/s23135894

**Published:** 2023-06-25

**Authors:** Ming-Chih Tsai, Edward T.-H. Chu, Chia-Rong Lee

**Affiliations:** 1 Computer Science and Information Engineering, National Yunlin University of Science and Technology, Yunlin 640301, Taiwan; m10917006@yuntech.edu.tw; 2 Bachelor Program in Interdisciplinary Studies, National Yunlin University of Science and Technology, Yunlin 640301, Taiwan; leecr@yuntech.edu.tw

**Keywords:** sitting posture recognition, pressure sensors, machine learning, embedded systems

## Abstract

Prolonged sitting with poor posture can lead to various health problems, including upper back pain, lower back pain, and cervical pain. Maintaining proper sitting posture is crucial for individuals while working or studying. Existing pressure sensor-based systems have been proposed to recognize sitting postures, but their accuracy ranges from 80% to 90%, leaving room for improvement. In this study, we developed a sitting posture recognition system called SPRS. We identified key areas on the chair surface that capture essential characteristics of sitting postures and employed diverse machine learning technologies to recognize ten common sitting postures. To evaluate the accuracy and usability of SPRS, we conducted a ten-minute sitting session with arbitrary postures involving 20 volunteers. The experimental results demonstrated that SPRS achieved an impressive accuracy rate of up to 99.1% in recognizing sitting postures. Additionally, we performed a usability survey using two standard questionnaires, the System Usability Scale (SUS) and the Questionnaire for User Interface Satisfaction (QUIS). The analysis of survey results indicated that SPRS is user-friendly, easy to use, and responsive.

## 1. Introduction

Currently, people spend a significant amount of time sitting on chairs while working or studying. Office workers and students typically spend more than 8–9 h per day sitting on a chair. This situation has worsened during and after the COVID-19 pandemic, as more people have been working from home. According to existing research [1], sitting in a poor posture on a chair for more than 6 h can cause bodily damage and increase health risks. For instance, it can elevate the risk of chronic diseases such as stroke, heart disease, diabetes, and metabolic syndrome. Poor sitting postures can also result in spine problems such as pelvis tucking, neck pain, uneven shoulders, and leg length discrepancy. Moreover, it can lead to bone injuries, sarcopenia, and poor circulation [2,3,4].

Many methods have been proposed to recognize sitting postures. They can be classified into three categories. The first category is to use wearable sensors to identify sitting postures [5,6,7,8,9,10]. Sangyong Ma et al. [10] used a three-axis accelerometer to collect the necessary data. After the data was normalized, support vector machine (SVM), and K-means algorithms were adopted to recognize a sitting posture. However, it is inconvenient for a user to wear a three-axis accelerometer for a long period of time. The second category is image recognition-based methods. A camera was first set in front of a seat or beside the seat to capture sitting postures. Then, an image recognition method was used to classify sitting postures [11,12,13,14,15,16,17]. For example, Lan Mu et al. [17] used a Webcam to recognize sitting postures. The Hausdorff distance was adopted to recognize face and body postures. However, their method can only identify the sitting posture of the upper body. The lower body cannot be identified. Moreover, the result of this method may be incorrect when someone passes by and is captured by the camera. The third category is pressure sensor-based methods. A matrix sensor mat or flexible pressure sensors were deployed on the chair to detect sitting postures. Haeyoon Cho et al. [18] deployed several pressure sensors on a chair cushion and two ultrasonic sensors on the back of a chair. The pressure sensors and ultrasound sensor were used together for data collection. The collected data was then used by Convolutional Neural Network (CNN) and Lower-Balanced Check Network (LBCNet) to classify sitting postures. However, this method required many pressure sensors, and the hardware cost was high.

In this paper, our primary objective is to design a system to recognize sitting postures and address the need for a cost-effective solution. We propose a pressure sensor-based system named SPRS (Sitting Posture Recognition System) to accurately recognize sitting postures. The hypotheses are that SPRS can capture, analyze, and distinguish sitting postures accurately. For this, we first deploy 25 pressure sensors uniformly on the hip area of the chair to collect pressure data. The collected data are used to identify the areas of the chair surface that capture key characteristics of sitting postures. Based on our results, the number of pressure sensors is reduced from 25 to 13, resulting in a low hardware cost. Next, we adopt different machine learning methods to recognize 10 common sitting postures and evaluate their performance. The machine learning methods used for evaluation include support vector machine (SVM), k-nearest neighbors (KNN), decision tree, random forest, and logistic regression. To have a comprehensive evaluation of the accuracy and usability of SPRS, we invite 20 volunteers to sit on the chair for ten minutes with arbitrary postures. According to our experiment results, the accuracy rate of SPRS in recognizing sitting postures is up to 99.1%. Additionally, we conduct a usability survey using two standard questionnaires: System Usability Scale (SUS) and Questionnaire for User Interface Satisfaction (QUIS). The SUS score is 86, indicating that SPRS is easy to use and responsive.

The rest of this paper is organized as follows. Section 2 discusses related works. Section 3 presents the system design and implementation. Section 4 analyzes the experimental results. Finally, Section 5 concludes this work and discusses future work.

## 2. Related Work

Many pressure sensor-based methods have been proposed in recent years. The main idea is to install pressure sensors on the hip area and back area of a chair to capture signals of sitting postures. Qisong Hu et al. [19] first installed six pressure sensors on the hips, armrests, and back areas of a chair to collect pressure readings. They then used a look-up table to build an Artificial Neural Network (ANN) model for classification. Their results showed an accuracy rate of 97.8% and a computation time of 9 ns. Wenyao Xu et al. [20] proposed a system using an electronic textile (eTextile) pressure sensor on the seat cushion to classify sitting postures. The eTextile consists of fibers coated with a conductive polymer that contains pressure and strain sensors. The system collected pressure signals when a user sat on the cushion. After filtering background noise, Dynamic Time Warping (DTW) was adopted to classify postures. Their experimental results showed an accuracy rate of only 85.9%. Jianquan Wang et al. [21] placed 81 pressure sensors on the hip of the chair and 90 pressure sensors on the back. They used Spiking Neural Network (Spiking-NN) to classify sitting postures, achieving an accuracy rate of 88.52%. However, their method requires sensor readings on the seat back, which can increase implementation cost. Zhe Fan, Qilong Wan, Xu Ran et al. [22,23,24] used a large array of pressure pads on the hip area to collect users’ hip pressure and converted the pressure signals into a pressure map. This pressure map was used to train a neural network model for classification. Although this method can correctly identify sitting postures, the hardware cost is relatively high, reaching USD 300 or more.

**Figure 1:**
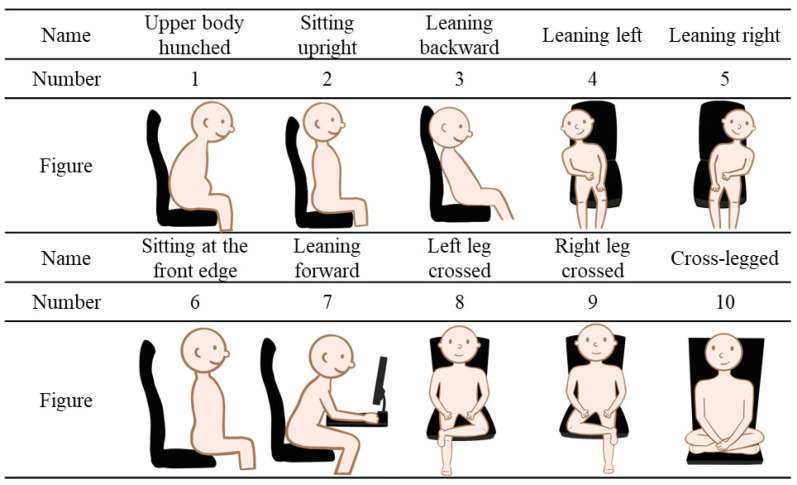
Illustrations of ten different sitting postures.

Some researchers have utilized hybrid sensor systems to recognize sitting postures. Haeseok Jeong et al. [25] proposed a hybrid sensor system consisting of six pressure sensors and six distance sensors placed on a chair. The collected pressure and distance readings were used to train a K-nearest neighbors (KNN) model for posture classification. Their results showed an accuracy of up to 92%. However, this method required the placement of distance sensors on the seat back, and the accuracy could be affected by users’ body size and height. Haeyoon Cho et al. [18] developed a system that combined two ultrasonic sensors and 16 pressure sensors. The collected signals were processed by an Arduino board and then transmitted to the Naver Cloud Platform, where Convolutional Neural Network (CNN) and Lower-Balanced Check Network (LBCNet) were used for posture classification. The recognition results were displayed on an Android phone. Although the system achieved an accuracy rate of up to 96%, it required 18 sensors, resulting in relatively high hardware costs. In contrast, our SPRS (Sitting Posture Recognition System) uses fewer pressure sensors while achieving similar performance. SPRS is capable of classifying ten different sitting postures, as shown in Figure 1. For ease of comparison, Table 1 summarizes the existing methods and SPRS. The detailed methodology and evaluation of SPRS are provided in Section 3 and Section 4.

## 3. Design and Implementation of the Sitting Posture Recognition System

### 3.1. Design Requirements and Challenges

The design requirements of SPRS include high accuracy, automation, low cost, and instant notification, all of which are described as follows. First, SPRS should be able to correctly classify different sitting postures. Second, SPRS needs to automatically detect poor sitting postures with minimum manual intervention. Third, the cost of SPRS should be affordable. Finally, SPRS should be able to notify the user when a poor posture has lasted longer than a predefined period of time. Since SPRS relies on a recognition method to determine the changes of sitting postures, the major difficulty is that different users may have very different sitting behaviors.

### 3.2. SPRS Overview

**Figure 2:**
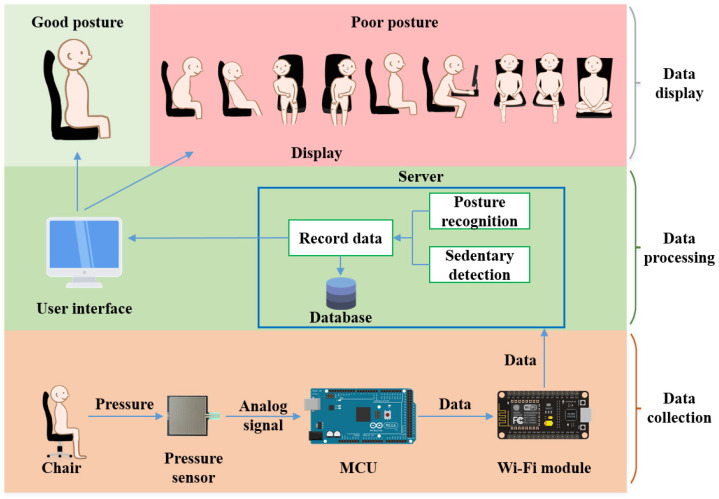
System architecture of SPRS.

The hardware architecture is shown in Figure 2. The first layer is the data collection layer, where the data from the pressure sensor set is digitized through an Analog-to-Digital (AD) converter before it can be processed by the Arduino. The Node MCU Wi-Fi module is utilized to establish a network connection and send the JSON format packet to the UDP Server in real-time. The second layer is the data processing layer. Upon receiving the data in JSON format, the computer stores it in the database. SPRS then processes the data and stores the recognition results back into the database. The third layer is the data display layer, which displays the recognition results and provides historical data to users. A demonstration video of SPRS is available online [26].

### 3.3. Smart Cushion Hardware

**Figure 3:**
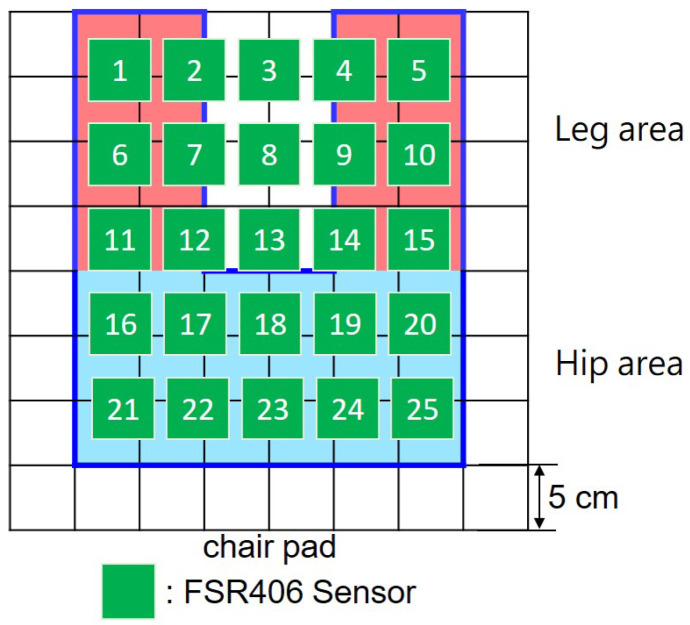
Preliminary deployment.

In order to minimize hardware costs, we initially installed 25 pressure sensors in the user’s sitting area, as depicted in Figure 3, to capture ten different sitting postures. These postures include upper body hunched, sitting upright (the correct sitting posture), leaning backward, leaning left, leaning right, sitting at the front edge, leaning forward, left leg crossed, right leg crossed, and both cross-legged postures. To further analyze the signals from the 25 pressure sensors, we used the random forest algorithm to perform feature selection. The algorithm extracts data into a training set using Bootstrap. The training set is then used to generate a decision tree, in which each node randomly selects features without repetition. We classify the dataset using these features and repeatedly generate different decision trees to form a forest. This allows us to determine the importance of each tree. Based on our experimental results, we have found that the random forest algorithm is the most effective way to recognize 10 sitting postures using fewer sensors in SPRS.

**Figure 4:**
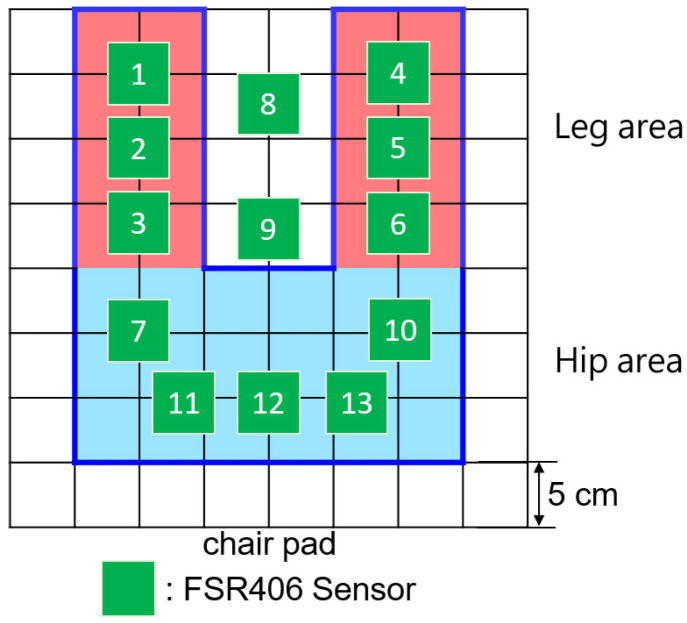
Final deployment.

Table 2 presents feature importance of random forest model. It shows that 13 out of the original 25 sensors significantly contributed to recognizing sitting postures, accounting for 94% of the feature weights. In order to maintain cost-effectiveness, we decided to retain only 13 sensors, as shown in Figure 4. In Section 4.8, we further evaluate the relationship between the accuracy and the number of sensors deployed on the cushion. Our experiment results confirmed that these selected sensors were sufficient for reliable classification, even though reducing the sensor count may have a slight impact on the resolution.

**Figure 5:**
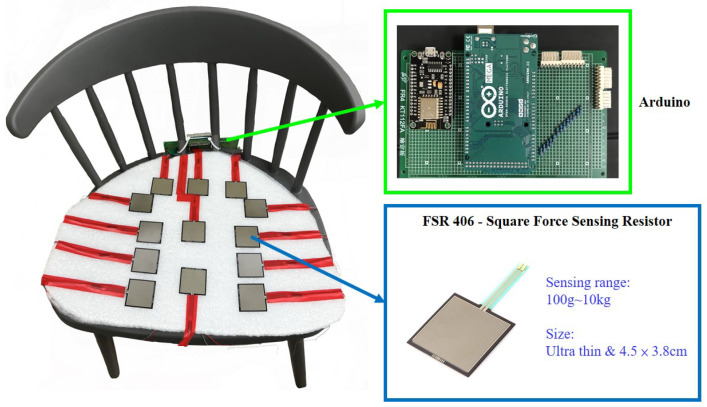
Hardware of smart cushion.

**Figure 6:**
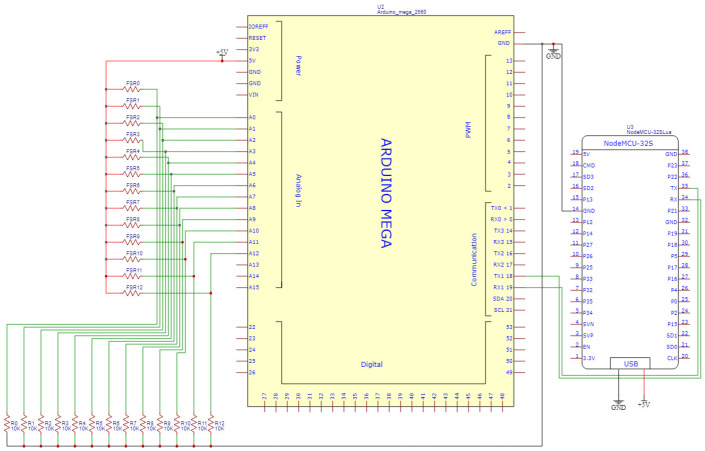
Circuit diagram of smart cushion.

As Figure 5 shows, the smart cushion is made up of 13 pressure sensors FSR-406, an Arduino Mega 2560 embedded evaluation board, and a NodeMCU Wi-Fi module. When SPRS was initially built, it was considered essential to accurately measure the pressure of the user’s hips and legs. Therefore, we opted for FSR-406, which exhibits lower resistance as the pressure increases. With an area of 4.5 × 3.8 cm and deployment on the PE foam board, the 13 sensors are capable of effectively detecting pressure. As Figure 6 illustrates, the FSR406 is a square, thin, resistive pressure sensor known for its low cost and high accuracy. It is capable of sensing pressure within the range of 0.1 kg to 10 kg, making it suitable for use in SPRS. The sensor’s resistance varies according to the applied pressure on its sensing area. Using the microcontroller’s 10-bit resolution AD (Analog-to-Digital) conversion module, the analog signal is converted into a digital signal. Subsequently, the pressure values for each area in the current sitting posture are calculated and transmitted to the computer via the Wi-Fi module.

### 3.4. Posture Recognition Methods

SPRS utilizes 13 pressure signals to classify sitting postures, aiming to assist users in determining the correctness of their sitting posture. Five different algorithms are employed by SPRS to identify sitting postures. The performance of each algorithm will be discussed in subsequent sections. The following sections will initially describe the data normalization preprocessing and then delve into the methods employed by different classification algorithms.

#### 3.4.1. Z-Score Standardization

Distance characteristics are commonly utilized by machine learning models such as SVM and KNN for prediction and classification purposes. These models classify features based on their distances, necessitating the standardization of data from different individuals and sitting postures to achieve a normal distribution with an average of 0 and a standard deviation of 1. In this study, we employed the Z-Score as the data preprocessing method [27], which is represented by the formula shown in Equation (1). Here, $x_{ij}$ represents the *j*-th pressure sensor value of the *i*-th sitting posture data, $z_{ij}$ represents the *j*-th pressure sensor value of the *i*-th sitting posture data after normalization, $\mu_{i}$ represents the average value of the *i*-th sitting posture data, and $\sigma_{i}$ represents the standard deviation of the *i*-th sitting posture data.

(1) $$z_{ij}=\frac{x_{ij}-\mu_{i}}{\sigma_{i}}.$$

#### 3.4.2. Support Vector Machine (SVM)

SVM is a commonly used classification method that exhibits excellent performance [28]. It determines the decision boundary for various sitting posture datasets and maximizes it into a hyperplane for classification. The number of sitting posture data points, denoted as *N*, can be expressed as $X=\left\{\left(x_{ij},y_{i}\right)\right\}$, where $x_{ij}$ represents the *j*-th pressure sensor value of the *i*-th sitting posture data, and $y_{i}$ represents the corresponding sitting posture category. The formula shown in (2) represents the good sitting posture $\left(y_{i}=1\right)$ and the hunchback posture $\left(y_{i}=-1\right)$ in SVM. In this formula, ω denotes the weight vector, and *b* represents the bias used to separate the hyperplane. The simplified formula is shown in (3). It enables the identification of different boundaries for each sitting posture.

(2) $$\left\{\begin{array}{c} \omega^{T}x_{ij}+b\geq 1\\ \omega^{T}x_{ij}+b\leq -1. \end{array}\right.$$

(3) $$y_{i}\left(\omega^{T}x_{ij}+b\right)\geq 1.$$

#### 3.4.3. K-Nearest Neighbor (KNN)

The concept of KNN is to find similar coordinates in the vector space, which also represents the distance between two sitting posture data points [29]. The Euclidean Distance is used to calculate the distance between different sitting posture data. Assuming there are two sitting posture data points in the training set, denoted as *n*, where $x_{i}$ represents the known sitting posture data: $x_{i}=\left[x_{1},x_{2},\cdots ,x_{13}\right]$, and $x_{j}$ represents the unknown sitting posture data: $x_{j}=\left[x_{1},x_{2},\cdots ,x_{13}\right]$. The Euclidean Distance between them can be expressed as Equation (4).

(4) $$d\left(x_{i},x_{j}\right)=\sqrt{\sum_{k=1}^{n}{\left(x_{ik}-x_{jk}\right)}^{2}}.$$

When calculating the distance, it is necessary to define the parameter *k*, which represents the number of nearest neighbors to consider. For instance, if *k* = 3, the algorithm will identify 3 coordinates with the shortest distance as neighbors and assign the sitting posture based on these 3 coordinates. The sitting posture that appears most frequently among these neighbors is considered the winning class. To achieve a complete classification among 10 sitting postures, *k* is usually chosen as an odd number to avoid ties. If *k* is too large, it may include irrelevant coordinates, leading to less accurate classification. On the other hand, if *k* is too small, it can result in overfitting. In this study, we utilized the characteristics of the KNN model, which involves placing a large number of sitting posture coordinates in a multi-dimensional space. When the sitting posture features are similar, the coordinates in the vector space also exhibit high similarity. This allows the KNN algorithm to classify the 10 sitting postures based on this shared feature.

#### 3.4.4. Decision Tree

The decision tree algorithm is a tree-structured algorithm that learns and derives decision rules to predict outcomes based on data features [30]. It is commonly used in regression and classification problems. The Gini coefficient is employed to determine the root node and construct the model in a recursive top-down manner until the dataset is completely partitioned. The Gini coefficient measures the impurity of a sample. The sitting posture dataset, denoted as *N*, can be represented as $X=\left\{\left(x_{ij},y_{i}\right)\right\}$, as shown in Equation (5). Here, *S* represents the number of labels, and $x_{ij}$ represents the probability of category $y_{i}$ appearing in the dataset *X*. A lower Gini coefficient indicates a lower level of impurity within the dataset. Since our study involves a multi-classification problem, it calculates 10 Gini coefficients for different sitting postures to perform the division.

(5) $$\mathrm{Gini}\left(X\right)=1-\sum_{y_{i}=1}^{S}x_{ij}^{2}.$$

The decision tree algorithm divides all the features and their corresponding values to determine the most suitable branches for classifying sitting postures. The depth of the tree reflects the complexity of the decision rules, and a deeper tree tends to capture more intricate patterns, making the model better aligned with real sitting postures. However, excessive noise in the data or an overly deep tree can lead to overfitting issues. To address this, we also adjusted the parameters related to tree depth, striking a balance between capturing meaningful patterns and preventing overfitting.

#### 3.4.5. Random Forest

Random forest is a machine learning algorithm that combines multiple decision trees to improve performance compared to a single decision tree [31]. Two conditions must be met for optimal results: first, each classifier must be different, and second, the accuracy of each classifier must exceed 50%. The key distinction between random forest and a single decision tree lies in the addition of the Bootstrap method to train the training set. The equation for multiple decision trees in the random forest is shown in Equation (5). The sitting posture dataset, denoted as *N*, can be represented as $X=\left\{\left(x_{ij},y_{i}\right)\right\}$, where *S* represents the number of labels, and $x_{ij}$ represents the probability of category $y_{i}$ appearing in the dataset *X*. A lower Gini coefficient indicates a lower level of impurity within the dataset. Once the 10 classification rules for sitting postures are generated, new data will be randomly input during calculation. Each decision tree in the forest will classify and produce a result. The forest will then select the most frequently occurring result as the final prediction. Although the Bootstrap method helps to mitigate overfitting, analyzing the results becomes more challenging due to the involvement of multiple decision trees.

#### 3.4.6. Logistic Regression

Logistic regression is derived from linear regression. It is a classification model that mainly calculates a decision boundary to divide the data [32]. It can also be called a linear classification model of regression, mainly using the sigmoid function to classify as shown in Equation (6). In Equation (6), the *N* sitting posture data can be represented as $X=\left\{\left(x_{ij},y_{i}\right)\right\}$, where $x_{ij}$ in *X* is the *j*-th pressure sensor value of the *i*-th sitting posture data, and $y_{i}$ is the sitting posture category. The *e* is a constant that represents the output value generated by g(X) and ranges from 0 to 1. The 10 sitting postures are classified according to a threshold.

(6) $$g\left(X\right)=\frac{1}{1+e^{-z}}.$$

However, logistic regression is usually used for binary classification. In our study, it generates *K* binary classifiers. When predicting, the sitting posture data will be put into the *K* classifiers, and the classifier with the highest prediction score wins.

### 3.5. Posture Recognition Application Software

#### 3.5.1. Software Architecture

**Figure 7:**
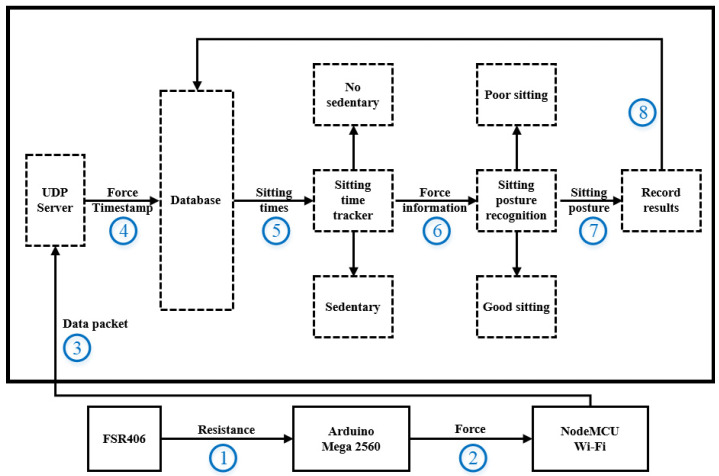
The architecture of posture recognition application software.

This section introduces the software architecture of SPRS, as shown in Figure 7. The data layer consists of five parts: UDP Server, Database, sitting time tracker, sitting posture recognition, and results. First, the Arduino Mega 2560 reads the voltage signal from the pressure sensor through an AD converter. It then converts the voltage signal into resistance values and calculates the Newton force (steps 1 to 2). Second, the NodeMCU Wi-Fi module encapsulates the converted Newton force data from all 13 pressure sensors into packets in JSON format and sends them to the computer once per second (step 3). Third, when the UDP Server receives the data sent by NodeMCU, it first parses the data and verifies whether the packet is lost or not. After verification, the data are recorded in the database along with the current time (step 4). The data are then extracted to determine if there is sedentary behavior. If sedentary behavior is detected, the system records the duration of sitting. If there is no sedentary behavior, the system continues to record the time (step 5). Next, machine learning algorithms are used to recognize the sitting posture based on the pressure data. Finally, the analysis results are sent back to the database and displayed on the screen.

#### 3.5.2. Software Interface

**Figure 8:**
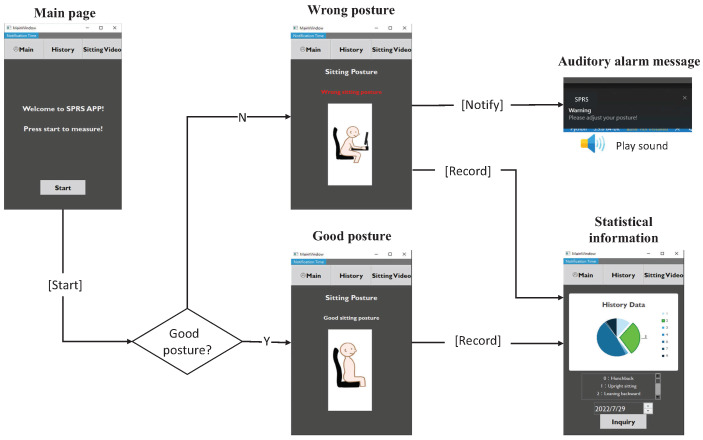
The wireflow of posture recognition application software.

In order to provide users with a clearer understanding of their current sitting posture, we have designed a user-friendly interface. The wireflow of the interface in shown in Figure 8. When the user presses the start button, it initiates a connection with the Wi-Fi module on the Arduino, and a progress bar is displayed on the screen. Once connected to the sitting posture recognition scene, an interface is presented that instantly displays the user’s sitting posture. During the recognition process, if the user maintains the correct sitting posture, SPRS will display the correct sitting posture on the screen. However, if the user’s sitting posture is poor, SPRS will display the current sitting posture as an incorrect sitting posture on the screen. Please note that users do not need to constantly visually monitor the app to determine if their posture deviates from a healthy position. There is an auditory alarm feature that notifies users if their posture has been incorrect for more than 30 min. SPRS also provides users with the ability to view historical records. On the historical data page, users can select the desired date to view the corresponding sitting posture data. Once a date is selected, the sitting postures recorded throughout the day are displayed in the form of a pie chart.

## 4. Experimental Results and Discussion

### 4.1. Experimental Design

To verify the accuracy of the model, we conducted a data collection process involving two stages. In the first stage, we collected data to train the model and evaluate its accuracy. In the second stage, we collected additional data to test the model’s classification performance and assess the usability of the system. The design of these experiments was inspired by renowned research studies [8,9,13,15,19,20,21,22,23,25]. It’s important to note that our experiment has received ethical approval from the Institutional Human Research Ethics Committee of National Chung Cheng University (CCUREC111051002). Each participant in the study was provided with detailed information about the experimental procedures and goals, and they voluntarily signed a research participant consent form.

#### 4.1.1. Stage One: Model Establishment

A total of six subjects participated in the study, consisting of three males and three females. The subjects’ characteristics were as follows: the height distribution ranged from 156 cm to 182 cm, the weight distribution ranged from 44 kg to 86 kg, and the age distribution ranged from 22 to 26 years old. During the experiment, the subjects were instructed to perform ten different sitting postures, which included upper body hunched, sitting upright (the correct sitting posture), leaning backward, leaning left, leaning right, sitting at the front edge, leaning forward, left leg crossed, right leg crossed, and both cross-legged postures (please refer to Figure 1). For each sitting posture, the subjects maintained the position for 1 min consecutively, resulting in a total test duration of 10 min. The data sampling rate is one sample per second, resulting in a total of 600 posture data points. Each data point includes the sensor readings of the 13 pressure sensors. Since we have six participants, there were 3600 data points used to establish models.

#### 4.1.2. Stage Two: Accuracy Validation

**Figure 9:**
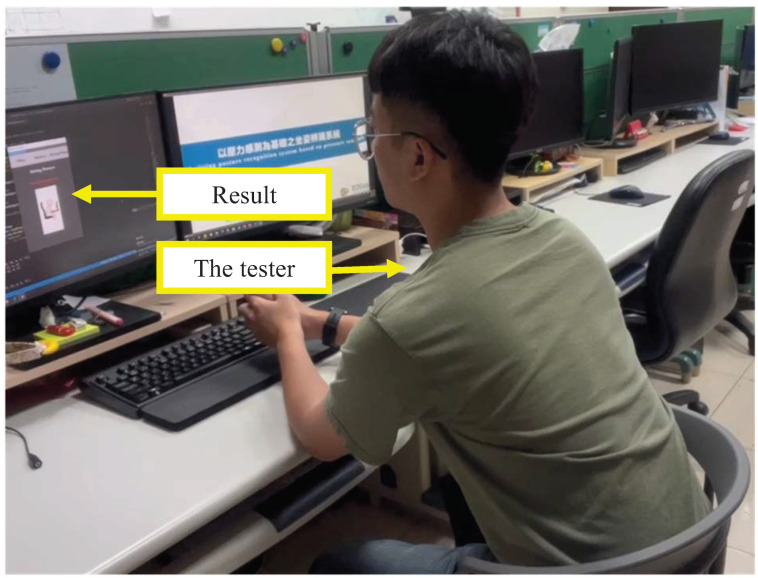
Sitting posture recognition by the tester.

In stage two, we recruited an additional 20 participants. There were 15 males and 5 females. The subjects’ characteristics included a height distribution ranging from 150 cm to 188 cm, a weight distribution ranging from 41 kg to 98 kg, and an age distribution ranging from 19 to 25 years old. Each participant allowed to freely change their sitting postures. Each sitting posture was lasted 30 s, and the total testing duration was 10 min for each participant. In addition, the data sampling rate was one sample per second resulting in a total of 600 posture samples. Each sample represents a posture classification result. Since we have twenty participants, there were 12,000 samples. In our experiment, all postures were represented and the number of each sitting posture was nearly equal. In order to obtain the ground truth of a user’s posture, the entire process was recorded by a camera. Figure 9 shows the camera’s view angle. Both the user’s posture and the classification result displayed on the monitor were recorded. To determine the accuracy, we reviewed the recorded videos and manually check the correctness of each classification result.

### 4.2. SVM Accuracy

**Figure 10:**
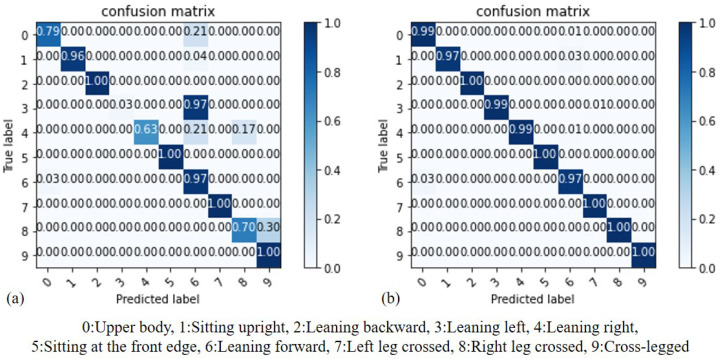
Accuracy comparison of SVM: (**a**) before standardization, (**b**) after standardization.

By comparing the results of SVM before and after standardization, we can evaluate whether standardization can effectively improve the accuracy of recognition. As depicted in Figure 10, (a) represents the results before standardization, while (b) represents the results after standardization. It can be observed that SVM, being a scaling model, is more sensitive to the distances between features. Consequently, training the SVM model on standardized data leads to improved results. As demonstrated in the aforementioned experiment, it is necessary to standardize the data for the SVM model before adjusting different Kernel Function parameters for comparison. The data used in this model comprises the subjects’ data from the second stage. The accuracy of the SVM model using different Kernel Functions is summarized in Table 3. The SVM model exhibits varying performance with different Kernel Functions. The parameters of the Kernel Function can be categorized into linear parameters and nonlinear parameters. In the case of linear parameters, the model achieves the highest accuracy rate of 99.1%. This is because the data are linearly separable, making the linear function the most accurate choice. On the other hand, the accuracy of the RBF Kernel Function is slightly lower at 98.4%.

### 4.3. KNN Accuracy

**Figure 11:**
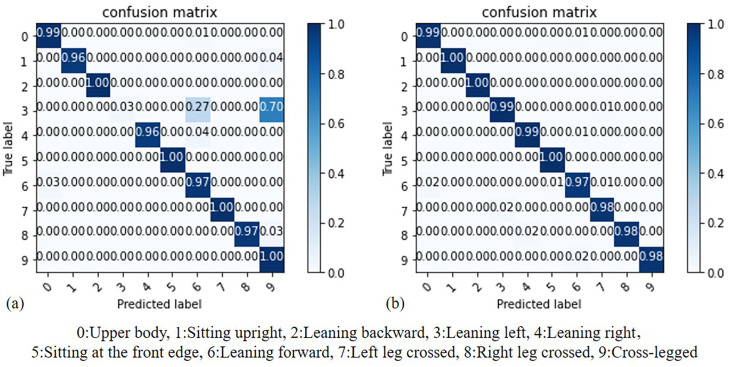
Accuracy comparison of KNN: (**a**) before standardization, (**b**) after standardization.

**Figure 12:**
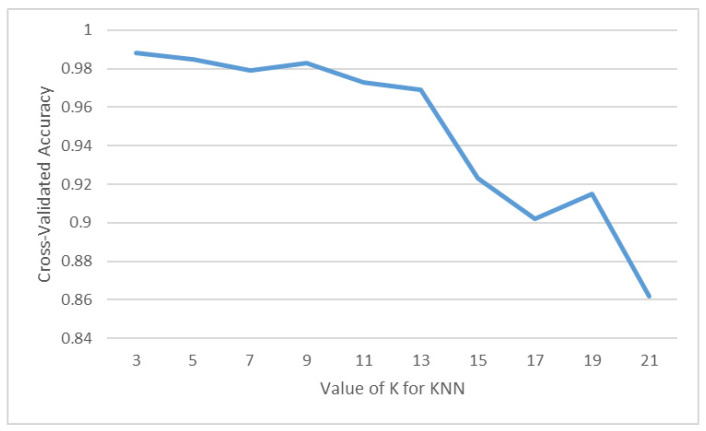
Performance of KNN with different K values.

By comparing the results of KNN before and after standardization, we can assess whether standardization truly enhances the accuracy of recognition. As illustrated in Figure 11, (a) represents the results before standardization, while (b) represents the results after standardization. It can be observed that KNN utilizes Euclidean distance to classify sitting postures and is therefore more sensitive to the distances between features. Consequently, the model trained on standardized data performs better, regardless of the chosen K value, with both K values of 3 yielding improved results. Based on the aforementioned experiment, it has been established that the KNN model’s data should be standardized prior to adjusting the K value for training. Since the K value directly influences the performance of KNN, it is crucial to compare different K values to determine the one with the highest accuracy. In Figure 12, the x-axis represents the value of K, while the y-axis represents the accuracy of the KNN model. Through this comparison, it can be observed that the K values of 1 and 3 yield the highest accuracy, reaching 98.8%.

### 4.4. Decision Tree Accuracy

**Figure 13:**
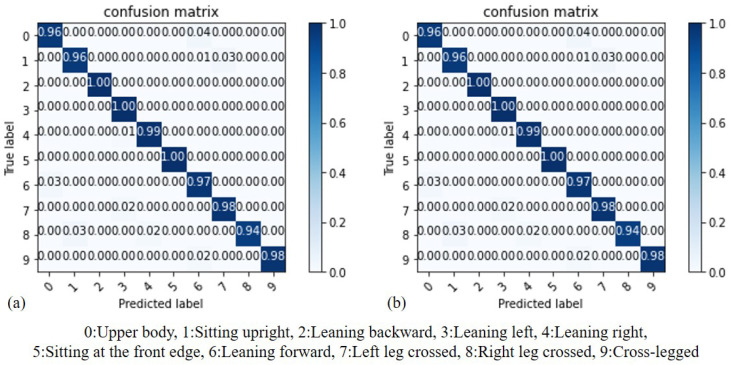
Accuracy comparison of DT: (**a**) before standardization, (**b**) after standardization.

By comparing the results of the Decision Tree (DT) model before and after standardization, we can assess whether standardization truly enhances the accuracy of recognition. As illustrated in Figure 13, (a) represents the results before standardization, while (b) represents the results after standardization. It can be observed that DT utilizes the Gini coefficient for node branch classification. In this case, the results before and after standardization are similar. Additionally, both models achieve their maximum accuracy when the Max_depth value is set to 10.

**Figure 14:**
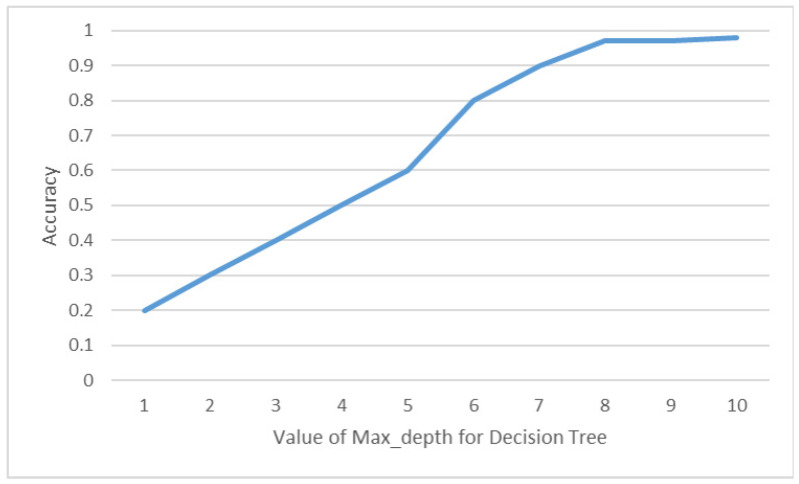
Accuracy of DT with different Max_depth values.

In our experiment, we conducted a comparison of model performance by adjusting the Max_depth value in the Decision Tree (DT) model. This parameter has a significant impact on the training performance of the model. In Figure 14, the x-axis represents the Max_depth value, while the y-axis represents the accuracy of the DT model. Through this comparison, it was determined that when the Max_depth value is set to 10, the model achieves the highest accuracy rate, which is 97.8%.

### 4.5. Random Forest Accuracy

**Figure 15:**
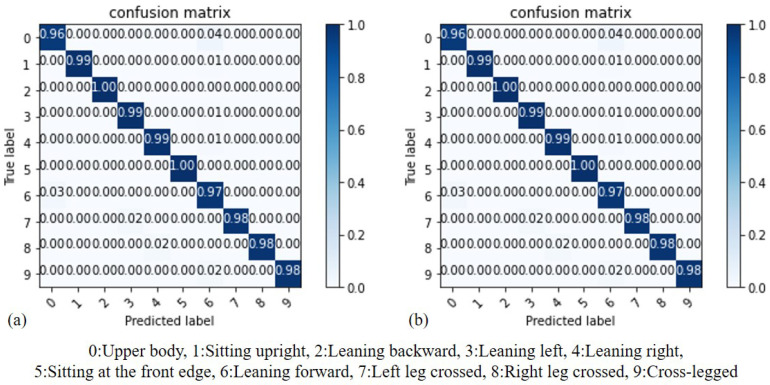
Accuracy comparison of RF: (**a**) before standardization, (**b**) after standardization.

**Figure 16:**
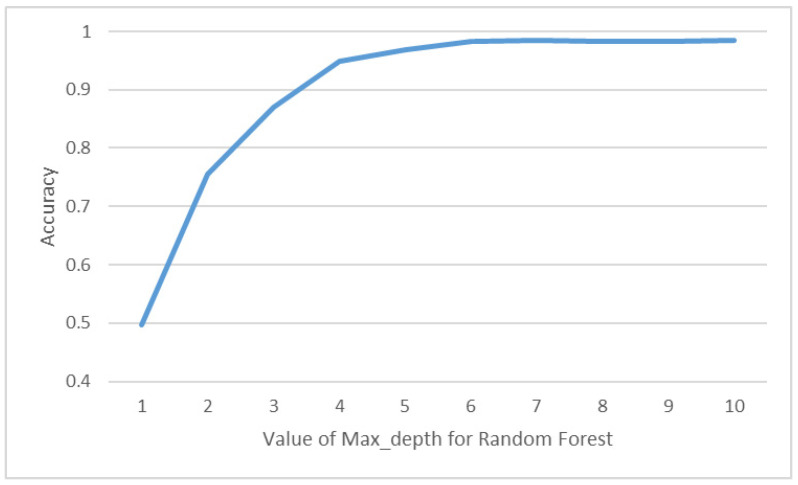
Accuracy of DT with different Max_depth value.

By comparing the results of random forest (RF) before and after standardization, we can assess whether standardization truly enhances the accuracy of recognition. As illustrated in Figure 15, (a) represents the results before standardization, while (b) represents the results after standardization. It can be observed that both RF and Decision Tree (DT) models are based on the Gini coefficient for node branch classification. Therefore, the results before and after standardization are similar. Additionally, both models achieve their maximum accuracy when the Max_depth value is set to 10. In our experiment, we compared the performances of the Random Forest (RF) model by adjusting the Max_depth value, considering its impact on both accuracy and computational cost. In Figure 16, the x-axis represents the Max_depth value, while the y-axis represents the accuracy of the RF model. Through this comparison, it was determined that when the Max_depth value is set to 7, the model achieves the highest accuracy rate, which is 98.4%.

### 4.6. Logistic Regression Accuracy

**Figure 17:**
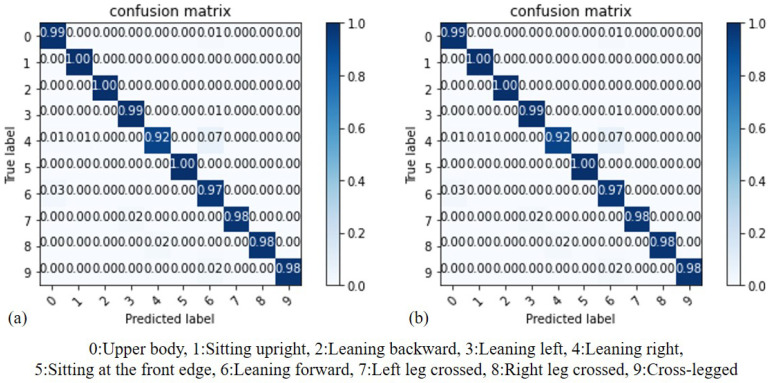
Accuracy comparison of LR: (**a**) before standardization, (**b**) after standardization.

By comparing the results of logistic regression (LR) before and after standardization, we can assess whether standardization truly enhances the accuracy of recognition. As shown in Figure 17, (a) represents the results before standardization, while (b) represents the results after standardization. It can be observed that LR calculates the probability of each classification based on probability theory and selects the class with the highest probability as the result. Therefore, the results after data standardization are similar to those before standardization. The accuracy rate remains at 98.1%.

### 4.7. Comparison of Algorithms

The analysis of the accuracy results in Table 4 reveals that all five machine learning algorithms achieved high accuracy rates of over 98%. Support vector machine (SVM) using the linear kernel attained the highest accuracy of 99.18%, indicating potential linear separability of the data. K-nearest neighbors (KNN) with K = 3 performed well, achieving an accuracy rate of 98.86%, suggesting clustering of similar sitting postures. Decision tree (DT) had a slightly lower accuracy of 97.83%, while random forest achieved an accuracy of 98.41%. Logistic regression (LR) showed a comparable accuracy of 98.19%. These results indicate the effectiveness of these algorithms in classifying and recognizing sitting postures, with SVM and KNN demonstrating particularly promising performance.

### 4.8. Influence of Sensor Placement on Accuracy

**Figure 18:**
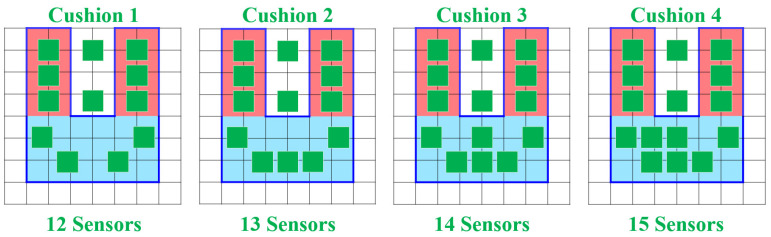
Four sensor placement configurations.

In order to lower the cost of the smart cushion, the number of pressure sensors was reduced from 25 to the first 15 sensors listed in Table 2. This study aimed to explore the impact of different sensor configurations on accuracy and cost. Four different sensor configurations were tested, namely 12, 13, 14, and 15 sensors, as illustrated in Figure 18. The objective was to assess the accuracies achieved with each configuration and determine the optimal balance between sensor count and cost.

Through the use of five classification methods (SVM, KNN, decision tree, random forest, and logistic regression), the first-stage subject data was used to train prediction models using four different sensor placement methods. The parameters for each algorithm were set without further adjustment: SVM used a Linear Kernel Function, KNN used a K value of 3, DT used a Max_depth value of 10, and RF used a Max_depth value of 7. The experiment revealed that when the number of pressure sensors was reduced to 13, all five algorithms maintained an accuracy rate of over 97%. Thus, the second sensor placement method was selected, eliminating the need to deploy sensors on the back area of the chair for recognition, as summarized in Table 5.

### 4.9. System Response Time

**Figure 19:**
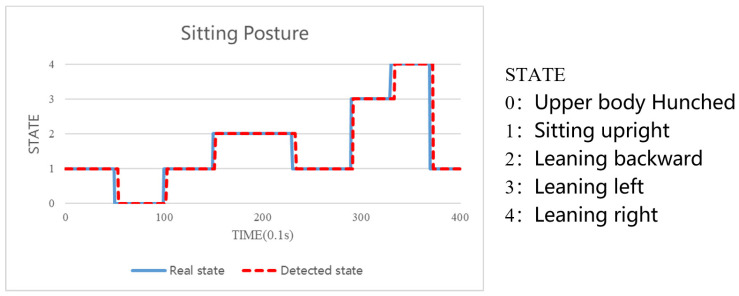
System response time.

In order to measure the response time of SPRS, the sitting posture images captured by the subjects in the second stage were manually compared with the real-time classification results of SPRS. The comparison results were then visualized in a line graph. The blue line represents the actual sitting posture recorded by the subjects, while the red line represents the sitting posture detected by SPRS. As depicted in Figure 19, the lines mostly overlap, indicating a close alignment between the actual and detected sitting postures. The minor non-overlapping portions can be attributed to a slight delay in the data transmission speed, resulting in a delay time of approximately 314 ms. However, this delay is imperceptible to users, and it is within an acceptable range considering that sitting durations exceeding 1 min are typically targeted for recognition.

### 4.10. Usability Analysis

The System Usability Scale (SUS), developed by John Brooke [33], is widely used to evaluate the usability of system interfaces, applications, and websites due to its objective, universal, repeatable, and quantifiable nature. It is suitable for small sample analysis, such as the sample of 20 participants in this study. The SUS questionnaire consists of ten questions, rated on a 5-point Likert scale. Odd-numbered questions are positive statements, and the score is obtained by subtracting 1 from the given score. Even-numbered questions are negative statements, and the score is calculated as 5 minus the given score. The total score is calculated by summing the converted scores of all questions and then multiplying by 2.5 to obtain the final SUS score. The overall SUS score of SPRS is 86, indicating a high level of usability, as shown in Table 6. Among the positive statements, Q5 received the highest percentage of strongly agree responses at 65.0%, indicating that SPRS excels in function integration. Among the negative statements, Q2 received the highest percentage of strongly disagree responses at 80.0%, suggesting that most users perceive the SPRS interface as not too complicated. Q9 and Q10 received lower scores, indicating that subjects may require more instruction or have difficulty using SPRS, highlighting areas for future improvement. Notably, among male participants, Q8 received the lowest score, suggesting that many of them do not appreciate constant reminders of incorrect sitting posture. Among female participants, Q4 received the lowest score, indicating a desire for instructional guidance before using SPRS, which will be addressed in future iterations of the system.

### 4.11. User Interface Satisfaction

The QUIS (Questionnaire for User Interface Satisfaction) is a widely used subjective satisfaction scale developed by the Human–Computer Interaction Lab (HCIL) at the University of Maryland, College Park [34]. It is employed to assess users’ satisfaction with SPRS (Smart Posture Recognition System). The questionnaire comprises six different aspects: overall reactions to the software, screen, terminology and system information, learning, system capabilities, and usability and user interface. Each aspect is rated on a 10-point scale, with 1 being the lowest score and 9 being the highest. Scores from 6 to 9 indicate satisfaction, scores from 4 to 5 indicate an average response, and scores from 0 to 3 indicate dissatisfaction. To ensure consistency between the questionnaire and SPRS, modifications were made, including the removal of questions related to voice in the system. The results of the QUIS (Questionnaire for User Interface Satisfaction) are presented in Table 7. The average score for the questionnaire is 7.68, with a standard deviation of 1.17. When considering scores from 6 to 9, it can be observed that 84.0% of users are satisfied with the interaction of SPRS and find it easy to operate. The aspect with the highest score is usability and user interface, indicating that many users are satisfied with SPRS and its notification interface, which aligns with the findings from the SUS questionnaire. The aspect with relatively lower scores is system capabilities, with Q25 receiving the lowest score. This suggests that users may require more guidance in using SPRS initially, highlighting an area for improvement. The largest difference between men and women in the questionnaire is seen in Q8 and Q26. Generally, men tend to have higher scores on these questions, while women have lower scores. This difference may stem from women’s focus on information presentation, layout, color, and ease of understanding in SPRS. Enhancements to the interface can be made to address these preferences in the future.

## 5. Conclusions

In this paper, a sitting posture recognition system based on pressure sensing is proposed. The system utilizes 13 pressure sensors to analyze the user’s sitting posture. Five algorithms are employed to compare the accuracies, and the results demonstrate the effectiveness of the SVM algorithm in identifying the user’s sitting posture. The system also exhibits a prompt response time, providing immediate feedback to the user regarding their current sitting posture and encouraging them to maintain good posture. With a high usability score of 86, participants express satisfaction with the real-time notification feature and find the user interface easy to operate. Furthermore, the system automatically records the user’s sitting posture, enabling users to review historical data and mitigate potential health issues arising from poor posture. In the future, we envision integrating SPRS with smart medical technology. By utilizing the user’s sitting posture data, the system could analyze the presence of conditions such as scoliosis or other spinal diseases, providing users with insights into their spinal health [2,35]. Furthermore, users could share their historical data with healthcare professionals, enabling them to gain a better understanding of the user’s past sitting posture and spinal health information. Different age groups of users will also be considered. In terms of hardware, our future plans involve reducing costs by designing a more simplified circuit and incorporating low-power microcontrollers to minimize battery charging time.

## Figures and Tables

**Table 1:** Methods for recognizing sitting posture using pressure sensors.

Reference	Sensor	Position of Sensor	Method	Accuracy Rate	Number of Sitting Postures (Figure 1)
Cho et al. [18] 2019	Pressure and ultrasonic sensors	Hip and back	LBCNet	96%	1, 2, 3, 4, 5, 8, 9
Wang et al. [21] 2021	Pressure sensor	Hip and back	SNN	88.50%	1, 2, 3, 4, 5, 6, 8, 9
Hu et al. [19] 2020	Pressure sensor	Hip, armrests and back	ANN	97.70%	2, 3, 4, 5, 8, 9
Fan et al. [22] 2022	Large pressure pad	Hip	CNN	99.80%	1, 2, 3, 4, 5
Wan et al. [23] 2021	Pressure pad	Hip	SVM	89.60%	2, 3, 4, 5
Ran et al. [24] 2021	Large pressure pad	Hip	RF	96.20%	1, 2, 3, 4, 5, 8, 9
Jeong et al. [25] 2021	Pressure and distance sensor	Hip and back	KNN	92%	1, 2, 3, 4, 5, 6
SPRS (This work)	Pressure sensor	Hip	SVM	99.18%	ALL

**Table 2:** Feature importance of random forest model.

Sensor ID	16	20	22	24	13
Weights	0.15	0.12	0.10	0.10	0.08
Sensor ID	11	15	10	1	3
Weights	0.08	0.07	0.07	0.05	0.04
Sensor ID	5	6	23	18	17
Weights	0.03	0.03	0.02	0.02	0.01

**Table 3:** Accuracy of four kernel functions.

Kernel Function	Accuracy
Linear	99.1%
Sigmoid	98.5%
RBF	98.4%
Polynomial	99.0%

**Table 4:** Accuracies of five ML algorithms.

Algorithms	Parameter	Accuracy
SVM	Linear	99.18%
KNN	K = 3	98.86%
Decision Tree	Max_depth = 10	97.83%
Random Forest	Max_depth = 7	98.41%
Logistic Regression	–	98.19%

**Table 5:** Accuracy comparison of four different sensor placements.

Algorithms	Cusion 1	Cusion 2	Cusion 3	Cusion 4
SVM	96.03%	99.18%	99.23%	99.23%
KNN	95.65%	98.86%	98.91%	98.91%
DT	94.25%	97.83%	97.89%	97.89%
RF	95.42%	98.41%	98.64%	98.64%
LR	94.89%	98.19%	98.53%	98.53%

**Table 6:** SUS questionnaire scores.

Question	Average	Standard Deviation	Percentage of Each Point of Likert’s 5-Point Scale (%)				
			1	2	3	4	5
Q1	4.44	0.73	0.0	0.0	15.0	30.0	55.0
Q2	1.22	0.44	80.0	20.0	0.0	0.0	0.0
Q3	4.44	0.73	0.0	0.0	5.0	40.0	55.0
Q4	1.67	0.71	45.0	40.0	15.0	0.0	0.0
Q5	4.78	0.44	0.0	0.0	5.0	30.0	65.0
Q6	1.44	0.53	40.0	55.0	5.0	0.0	0.0
Q7	4.56	0.53	0.0	0.0	0.0	45.0	55.0
Q8	1.44	0.53	50.0	45.0	5.0	0.0	0.0
Q9	4.22	0.83	0.0	0.0	10.0	40.0	50.0
Q10	1.78	0.67	35.0	40.0	20.0	5.0	0.0

**Table 7:** The scores of QUIS.

Aspects	Question	Average	Average Score of Questions	Standard Deviation	Percentage of Each Point of 10-Point Scale (%)									
					0	1	2	3	4	5	6	7	8	9
Overall reactions to the software	Q1	7.75	7.53	0.91	0.0	0.0	0.0	0.0	0.0	0.0	10.0	25.0	45.0	20.0
	Q2	7.45		1.93	0.0	5.0	0.0	0.0	0.0	5.0	10.0	20.0	25.0	35.0
	Q3	7.10		1.48	0.0	0.0	5.0	0.0	0.0	0.0	20.0	25.0	45.0	5.0
	Q4	7.75		1.37	0.0	0.0	0.0	0.0	5.0	0.0	10.0	25.0	20.0	40.0
	Q5	7.60		1.23	0.0	0.0	0.0	0.0	0.0	5.0	15.0	25.0	25.0	30.0
Screen	Q6	8.15	7.76	1.04	0.0	0.0	0.0	0.0	0.0	0.0	10.0	15.0	25.0	50.0
	Q7	7.70		0.98	0.0	0.0	0.0	0.0	0.0	0.0	15.0	20.0	45.0	20.0
	Q8	7.45		1.05	0.0	0.0	0.0	0.0	0.0	5.0	15.0	20.0	50.0	10.0
	Q9	7.65		0.93	0.0	0.0	0.0	0.0	0.0	0.0	10.0	35.0	35.0	20.0
Terminology and system information	Q10	7.25	7.73	1.21	0.0	0.0	0.0	0.0	0.0	5.0	25.0	30.0	20.0	20.0
	Q11	7.75		1.12	0.0	0.0	0.0	0.0	0.0	5.0	5.0	30.0	30.0	30.0
	Q12	8.20		0.89	0.0	0.0	0.0	0.0	0.0	0.0	5.0	15.0	35.0	45.0
	Q13	7.80		1.06	0.0	0.0	0.0	0.0	0.0	0.0	10.0	35.0	20.0	35.0
	Q14	8.05		1.19	0.0	0.0	0.0	0.0	0.0	5.0	10.0	5.0	35.0	45.0
	Q15	7.35		1.57	0.0	0.0	0.0	5.0	0.0	5.0	15.0	20.0	30.0	25.0
Learning	Q16	8.05	7.77	1.1	0.0	0.0	0.0	0.0	0.0	5.0	0.0	25.0	25.0	45.0
	Q17	7.45		1.23	0.0	0.0	0.0	0.0	0.0	10.0	10.0	25.0	35.0	20.0
	Q18	7.65		1.04	0.0	0.0	0.0	0.0	0.0	0.0	15.0	30.0	30.0	25.0
	Q19	7.80		1.01	0.0	0.0	0.0	0.0	0.0	0.0	10.0	30.0	30.0	30.0
	Q20	7.95		1.1	0.0	0.0	0.0	0.0	0.0	0.0	10.0	30.0	15.0	45.0
	Q21	7.70		0.98	0.0	0.0	0.0	0.0	0.0	0.0	15.0	20.0	45.0	20.0
System capabilities	Q22	7.50	7.53	0.95	0.0	0.0	0.0	0.0	0.0	0.0	15.0	35.0	35.0	15.0
	Q23	7.85		1.14	0.0	0.0	0.0	0.0	0.0	5.0	5.0	25.0	30.0	35.0
	Q24	7.40		1.19	0.0	0.0	0.0	0.0	0.0	5.0	20.0	25.0	30.0	20.0
	Q25	7.40		1.6	0.0	0.0	0.0	5.0	0.0	0.0	25.0	20.0	15.0	35.0
Usability and user interface	Q26	7.55	7.76	1.36	0.0	0.0	0.0	5.0	0.0	0.0	5.0	30.0	40.0	20.0
	Q27	7.75		0.91	0.0	0.0	0.0	0.0	0.0	0.0	15.0	10.0	60.0	15.0
	Q28	7.85		1.23	0.0	0.0	0.0	0.0	0.0	5.0	10.0	20.0	25.0	40.0
	Q29	7.70		0.98	0.0	0.0	0.0	0.0	0.0	5.0	0.0	35.0	40.0	20.0
	Q30	7.95		1.19	0.0	0.0	0.0	0.0	0.0	5.0	10.0	10.0	35.0	40.0
ALL		7.68		1.17	0.0	0.0	0.0	1.0	0.0	3.0	12.0	24.0	32.0	28.0

## Data Availability

Data available upon request.
